# Supplementary material for: T- and B-Cells in the Inner Invasive Margin of Hepatocellular Carcinoma after Resection Associate with Favorable Prognosis
Source: Cancers (Basel). 2022 Jan 25;14(3):604. doi: 10.3390/cancers14030604 (PMC8833821; doi:10.3390/cancers14030604)
Supplement: Supplementary file 1 [file cancers-14-00604-s001.zip › cancers-1554696-supplementary.pdf]

Supplementary data

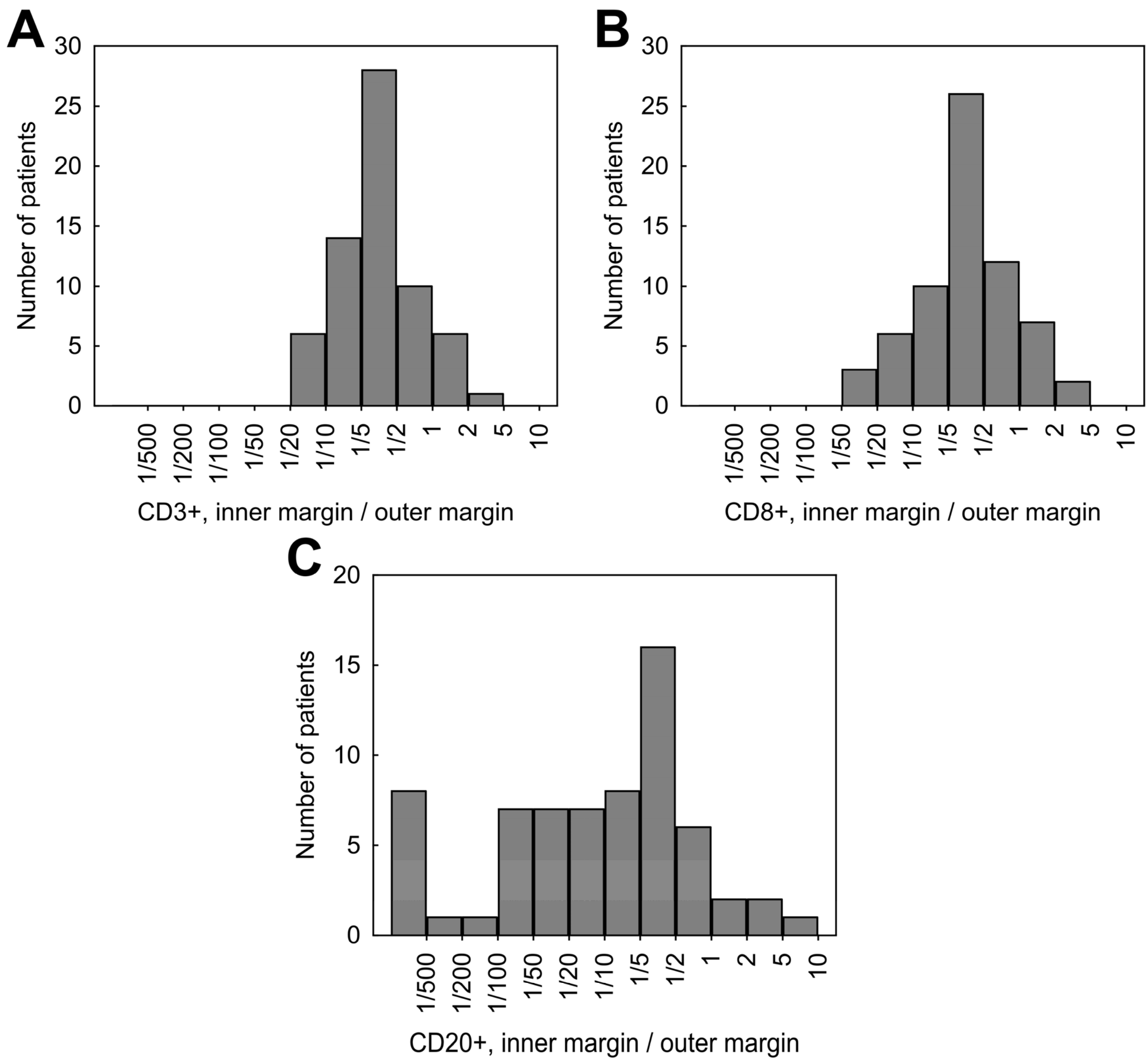

Figure S1. Distribution of the patients according to the inner M/outer M ratios of nucleated profiles of tumor infiltrating lymphocytes. CD3+ (A), CD8+ (B), CD20+ (C) (see also Table 2).

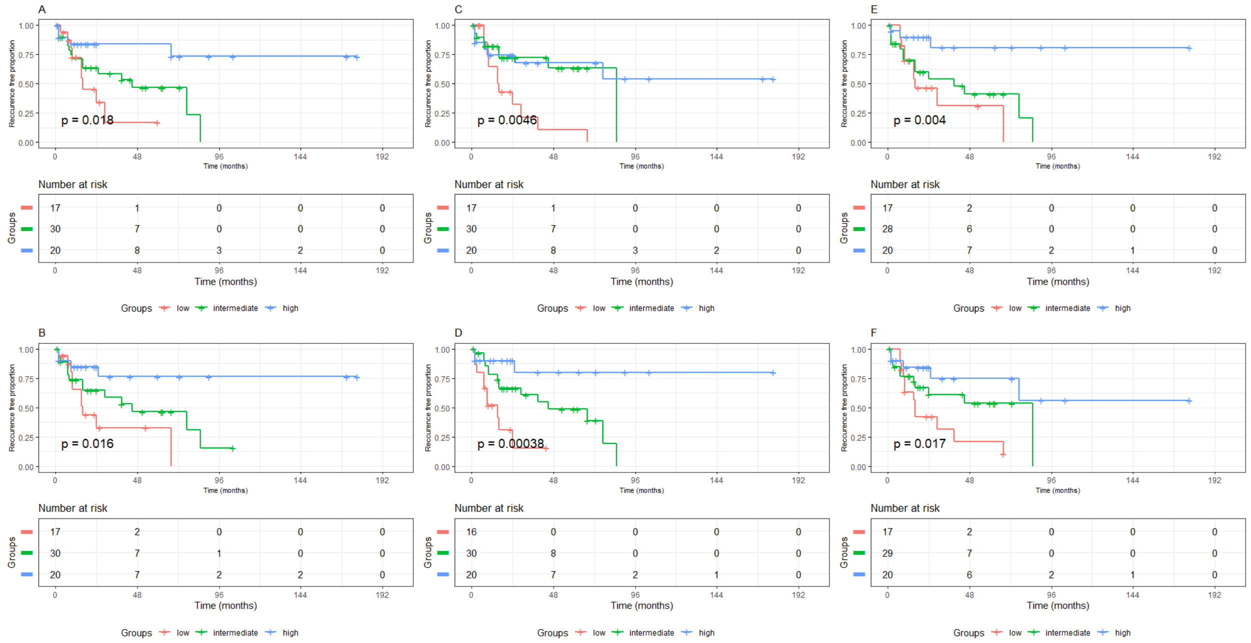

Figure S2. Kaplan-Meier analysis for time to recurrence according to low vs intermediate vs high densities of nucleated profiles of tumor infiltrating lymphocytes in the tumor center (A-C) and inner margin (D-F).

A – CD20+ B cells, B – CD3+ T cells, C – CD8+ T-cells, D – CD20+ B cells, E – CD3+ T cells, F – CD8+ T-cells. Statistical differences assessed with Log-rank test.

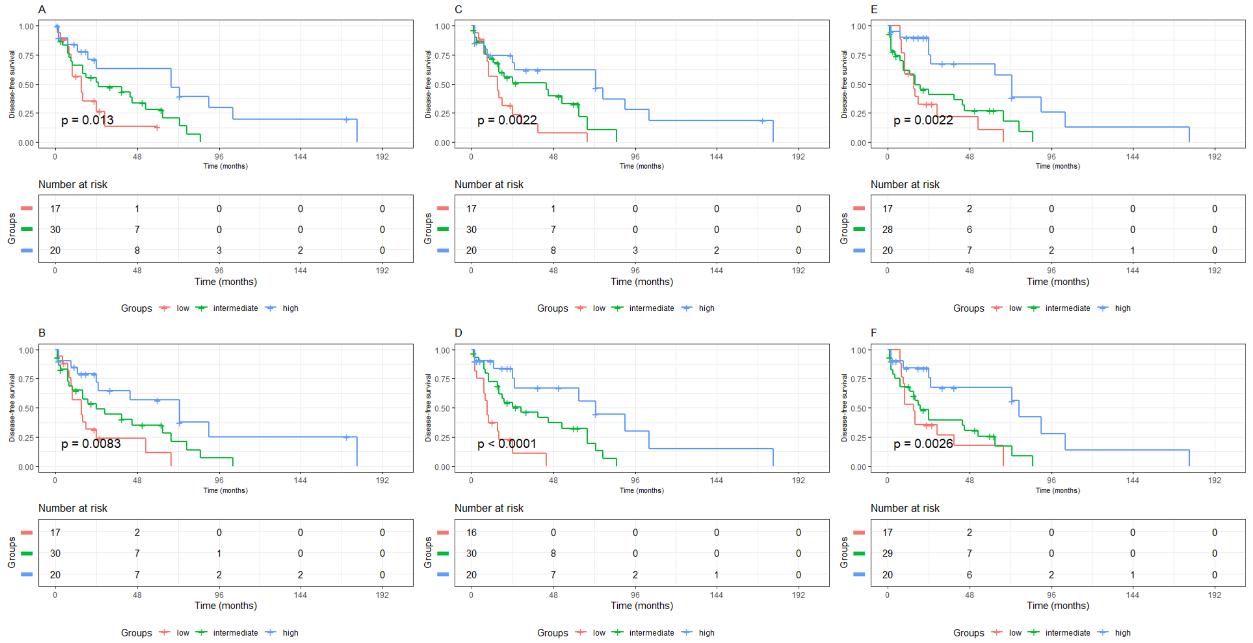

Figure S3. Kaplan-Meier analysis for disease-free survival according to low vs intermediate vs high densities of nucleated profiles of tumor infiltrating lymphocytes in the tumor center (A-C) and inner margin (D-F).

A – CD20+ B cells, B – CD3+ T cells, C – CD8+ T-cells, D – CD20+ B cells, E – CD3+ T cells, F – CD8+ T-cells. Statistical differences assessed with Log-rank test.

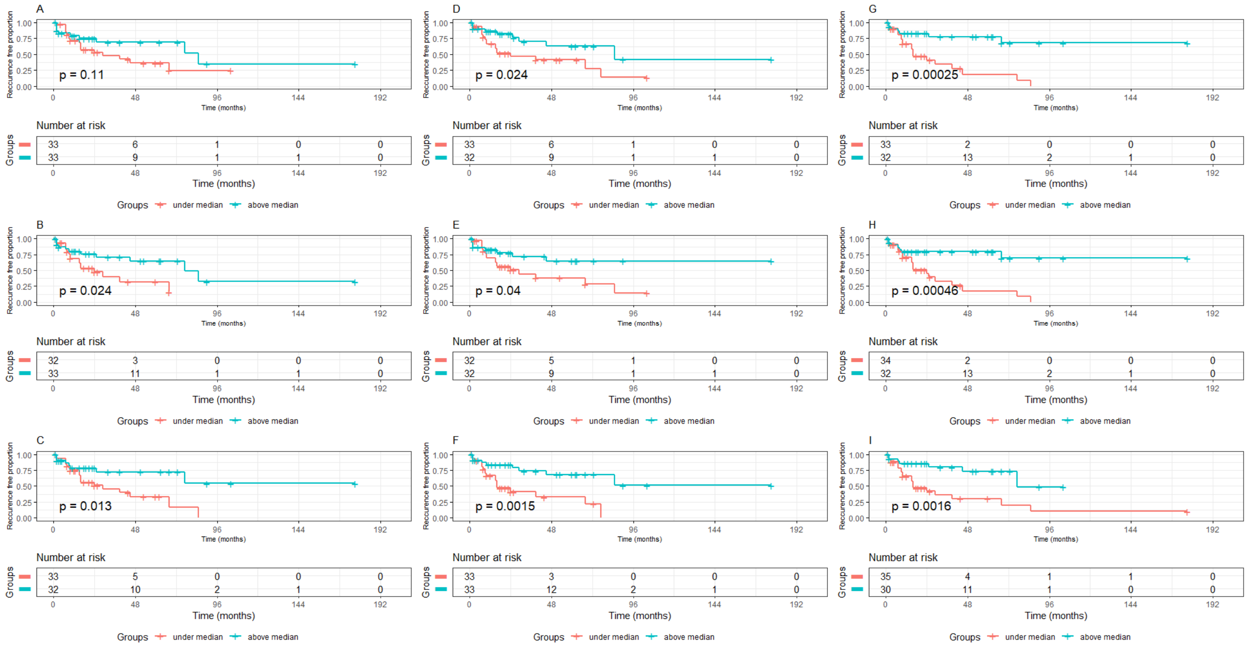

Figure S4. Kaplan-Meier analysis for time to recurrence according to above-median vs under-median ratios between densities of nucleated profiles of tumor infiltrating lymphocytes in different regions of interest.

A – CD8+ T-cells inn M/PT, B – CD3+ T-cells inn M/out M, C – CD8+ T-cells TC/PT, D – CD3+ T-cells TC/out M, E – CD3+ T-cells TC/PT, F – CD20+ B cells inn M/out M, G – CD20+ B cells TC/out M, H – CD20+ B cells TC/M, I – CD20+ B cells TC/PT. Statistical differences assessed with Log-rank test.

Abbreviations: TC: center of the tumor, PT: peritumor liver, M: invasive margin, inn M: inner invasive margin, out M: outer invasive margin

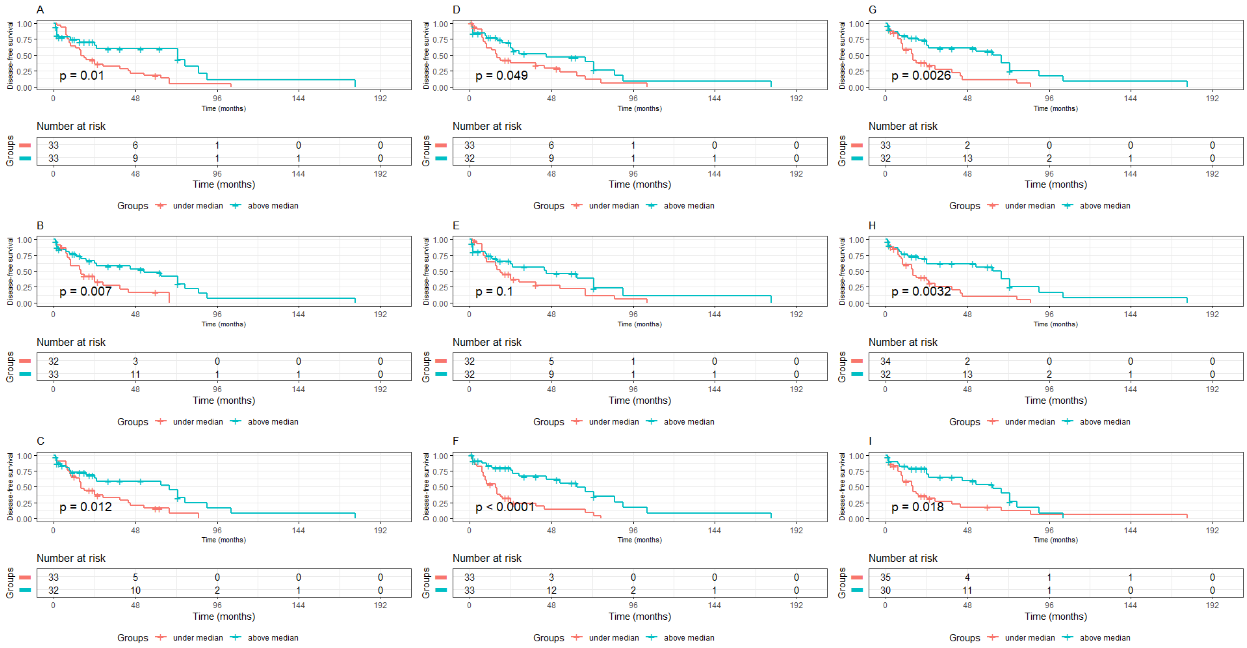

Figure S5. Kaplan-Meier analysis for disease-free survival according to above-median vs under-median ratios between densities of nucleated profiles of tumor infiltrating lymphocytes in different regions of interest.

A – CD8+ T-cells inn M/PT, B – CD3+ T-cells inn M/out M, C – CD8+ T-cells TC/PT, D – CD3+ T-cells TC/out M, E – CD3+ T-cells TC/PT, F – CD20+ B cells inn M/out M, G – CD20+ B cells TC/out M, H – CD20+ B cells TC/M, I – CD20+ B cells TC/PT. Statistical differences assessed with Log-rank test.

Abbreviations: TC: center of the tumor, PT: peritumor liver, M: invasive margin, inn M: inner invasive margin, out M: outer invasive margin

Table S1. Clinical backgrounds of enrolled hepatocellular carcinoma patients

**Variables**	
No. of patients	67
Age, y (median, min-max)	69 (24-86)
Sex (male/female), n (%)	52/15 (77.6%/22.4%)
Background disease, n (%)	
Cirrhosis	15 (22.4%)
Hepatitis	42 (62.7%)
Hepatitis C	3 (4.5%)
Alcoholic steatohepatitis	7 (10.4%)
Cryptogenic chronic hepatitis	7 (10.4%)
Non-alcoholic steatohepatitis	16 (23.9%)
Mixed etiology of hepatitis	9 (13.4%)
NAFLD	2 (3.0%)
Unknown	8 (11.9%)
Diabetes Mellitus; yes/no, n (%)	28/49 (41.8%/58.2%)
Metabolic syndrome; yes/no/unknown	10/29/28 (14.9%/43.3%/41.8%)
Child-Pugh score, n (%)	
A	62 (92.5%)
B	3 (4.5%)
unknown	2 (3.0%)
Gross structure of the tumor, n (%)	
single distinctly nodular	42 (62.7%)
multinodular	17 (25.4%)
vaguely nodular	6 (9.0%)
infiltrative	1 (1.5%)
Tumor number; solitary/multiple, n (%)	58/9 (86.6%/13.4%)
Tumor size, n (%)	
≤ 5 cm	30 (44.8%)
> 5 cm	34 (50.7%)
unknown	3 (4.5%)
Tumor stage, TNM, n (%)	
I	46 (68.7%)
II	13 (19.4%)
III	5 (7.5%)
IV	3 (4.5%)
Alpha-fetoprotein, IU/ml (median, min-max)	4.0 (1.0-6612.0)
Events, n (%)	
Recurrence	29 (41.8%)
Death	38 (56.7%)

Abbreviations: NAFLD: non-alcoholic fatty liver disease

Table S2. Histopathological features of hepatocellular carcinoma

**Variables**	
Tumor grade, Edmondson-Steiner, n (%)	
1	0 (0%)
2	47 (70.1%)
3	18 (26.9%)
4	2 (3.0%)
Tumor grade, WHO, n (%)	
1	32 (47.8%)
2	33 (49.3%)
3	2 (3.0%)
4	0 (0%)
Growth type, n (%)	
desmoplastic	31 (46.3%)
infiltrative	2 (3.0%)
pushing	0 (0%)
mixed (desmoplastic, infiltrative, pushing)	34 (50.7%)
Encapsulation (0/1/2/3), n (%)	1/9/26/31 (1.5%/13.4%/38.8%/46.3%)
Amount of stromal component, n (%)	
0	14 (20.9%)
1	27 (40.3%)
2	14 (20.9%)
3	12 (17.9%)
Cytological variants, n (%)	
Typical (hepatocyte-like)	48 (71.6%)
Clear cells	4 (6.0%)
Steatohepatitis-like	3 (4.5%)
Mixed	12 (17.9%)
Architectural grade, n (%)	
Microtrabecular, grade I	16 (23.9%)
Pseudoglandular, grade II	15 (22.4%)
Midtrabecular, grade III	28 (41.8%)
Macrotrabecular and solid/bizarre, grade IV	8 (11.9%)
Nuclear grade, n (%)	
I	18 (26.9%)
II	34 (50.7%)
III	14 (20.9%)
IV	1 (1.5%)
Nucleolar grade, n (%)	
I	17 (25.4%)
II	25 (31.3%)
III	25 (31.3%)
IV	0 (0%)
Tumor micronodularity, yes/no, n (%)	28/49 (41.8%/58.2%)
Microvascular invasion, yes/no, n (%)	18/49 (26.9%/43.1%)
Microsatellites, yes/no, n (%)	30/37 (44.8%/55.2%)
Tumor necrosis, yes/no, n (%)	27/50 (40.3%/59.7%)

Table S3. The estimated probability of outcomes in Kaplan-Meier analysis

	1 year	3 years	5 years
RFP	74.6% (63.6%-85.6%)	55.9 % (41.9%-69.9%)	48.7% (33.2%-64.4%)
DFS	67.6% (56.1%-79.1%)	43.2 % (30.1%-56.2%)	33.2% (20.0%-46.5%)
OS	88.1% (80.1%-96.1%)	70.6 % (58.5%-82.6%)	49.4% (35.1%-63.6%)

RFP: recurrence-free proportion
DFS: disease-free survival
OS: overall survival

Table S4. Univariable analysis of clinical and pathology variables associated with time to recurrence (TTR) and disease-free survival (DFS)

	TTR			DFS		
	HR	95% CI	P	HR	95% CI	P
Growth type, n=67 (continuous)	1.01	0.73-1.39	0.978	1.06	0.83-1.35	0.647
Extent of encapsulation, n=67 (continuous)	1.25	0.72-2.17	0.435	0.94	0.63-1.39	0.739
Microvascular invasion, n=67 (yes vs no)	2.06	0.96-4.40	0.063	1.59	0.85-2.97	0.146
Diabetes mellitus, n=67 (yes vs no)	2.07	0.98-4.39	0.058	1.66	0.91-3.01	0.097
Viral hepatitis, n=67 (yes vs no)	0.66	0.19-2.26	0.505	0.46	0.16-1.31	0.145
Gender, n=67 (males vs females)	0.89	0.36-2.21	0.807	0.86	0.42-1.74	0.672
Age, n=67 (continuous)	0.96	0.93-0.99	**0.007**	0.98	0.95-1.01	0.106
Tumor Size, n=67 (continuous)	0.76	0.42-1.41	0.387	0.85	0.53-1.36	0.489
Tumor grade (WHO) n=67 (continuous)	0.78	0.38-1.59	0.492	0.71	0.40-1.24	0.228
Tumor grade (Edmondson-Steiner), n=67 (continuous)	0.57	0.24-1.36	0.205	0.70	0.37-1.31	0.266
Architectural grade, n=67 (continuous)	1.06	0.73-1.53	0.767	0.92	0.70-1.22	0.570
Stromal component, n=67 (continuous)	1.04	0.69-1.58	0.847	1.04	0.75-1.46	0.803
Cytological type, n=67 (continuous)	1.34	0.62-2.90	0.464	1.24	0.67-2.32	0.496
Nuclear grade, n=67 (continuous)	0.70	0.42-1.15	0.155	0.74	0.50-1.09	0.125
Nucleolar grade, n=67 (continuous)	0.83	0.51-1.36	0.464	0.92	0.63-1.35	0.678
Necrosis, n=67 (yes vs no)	0.88	0.39-1.96	0.752	1.25	0.69-2.26	0.464
Tumor micronodularity, n=67 (yes vs no)	0.87	0.40-1.91	0.736	1.04	0.57-1.90	0.888
Microsatellites, n=67 (yes vs no)	1.09	0.50-2.35	0.831	1.37	0.76-2.47	0.303
TNM stage, n=67 (continuous)	1.50	1.04-2.16	**0.032**	1.24	0.90-1.71	0.185
Alpha-fetoprotein, n=46 (continuous)	1.00	1.00-1.00	0.880	1.00	1.00-1.00	0.408
Child-Pugh score, n=65 (A vs B)	1.19	0.16-8.86	0.867	1.81	0.55-5.95	0.332

“No”, female gender or “A” were the reference categories for dichotomous variables.

Bold values indicate statistical significance at the P <0.05 level.

Abbreviations: HR, hazard ratio; CI, confidence interval; TTR, time to recurrence; DFS, disease-free survival

Table S5. Densities of nucleated profiles of tumor infiltrating lymphocytes (Q_A) per individual ROI associated with time to recurrence and disease-free survival (univariable analysis) in 64 most recent patients

	Q_A	TTR			DFS		
		**HR**	**95% CI**	**P**	**HR**	**95% CI**	**P**
**Tumor center**							
**CD3,** n=64	int vs low	0.60	0.26-1.40	0.235	0.63	0.31-1.27	0.197
**P=0.036*, 0.022†**	high vs low	0.21	0.06-0.68	**0.010**	0.29	0.12-0.70	**0.006**
**CD8,** n=64	int vs low	0.29	0.12-0.72	**0.008**	0.50	0.25-1.00	0.075
**P=0.011*, 0.013†**	high vs low	0.30	0.11-0.82	**0.019**	0.28	0.12-0.66	**0.004**
**CD20**, n=64	int vs low	0.57	0.24-1.36	0.204	0.62	0.30-1.29	0.197
P=0.067*, 0.068†	high vs low	0.23	0.07-0.80	**0.020**	0.33	0.13-0.84	**0.020**
**Inner invasive margin**							
**CD3,** n=62	int vs low	0.69	0.29-1.64	0.404	0.71	0.35-1.45	0.353
**P=0.041*, 0.028†**	high vs low	0.18	0.05-0.69	**0.012**	0.30	0.12-0.73	**0.008**
**CD8**, n=63	int vs low	0.45	0.19-1.07	0.071	0.72	0.37-1.43	0.353
P=0.052*, **0.033†**	high vs low	0.29	0.10-0.87	**0.027**	0.29	0.11-0.74	**0.009**
**CD20**, n=63	int vs low	0.33	0.14-0.79	**0.013**	0.32	0.15-0.67	**0.002**
**P=0.004*, 0.001†**	high vs low	0.12	0.03-0.46	**0.002**	0.17	0.06-0.44	**<0.001**
**Outer invasive margin**							
**CD3,** n=62	int vs low	0.54	0.21-1.42	0.212	0.52	0.24-1.13	0.098
P=0.435*, 0.254†	high vs low	0.62	0.23-1.73	0.363	0.70	0.32-1.55	0.377
**CD8,** n=63	int vs low	0.50	0.20-1.27	0.145	0.46	0.22-0.96	**0.038**
P=0.328*, 0.083†	high vs low	0.58	0.21-1.61	0.292	0.47	0.20-1.08	0.073
**CD20,** n=63	int vs low	0.79	0.32-1.96	0.605	0.82	0.40-1.69	0.588
P=0.714*, 0.667†	high vs low	0.66	0.24-1.81	0.416	0.69	0.30-1.56	0.370
**Peritumor liver**							
**CD3,** n=61	int vs low	0.42	0.15-1.16	0.095	0.67	0.30-1.49	0.324
P=0.141*, 0.216†	high vs low	1.05	0.41-2.73	0.916	1.27	0.56-2.87	0.566
**CD8,** n=62	int vs low	0.86	0.34-2.20	0.780	1.18	0.56-2.49	0.661
P=0.759*, 0.889†	high vs low	1.22	0.45-3.26	0.710	1.19	0.52-2.76	0.692
**CD20,** n=62	int vs low	0.20	0.07-0.56	**0.009**	0.39	0.18-0.85	**0.017**
**P=0.003*, 0.005†**	high vs low	1.05	0.43-2.60	0.900	1.37	0.62-3.04	0.439

For all cells and regions of interest the raw densities of nucleated profiles of CD3+, CD8+ and CD20+ tumor infiltrating lymphocytes per area section (mm^2) were converted into percentiles and then categorized into low (0-25 percentile), intermediate (25-70 percentile) or high (70-100 percentile).

Hazard ratios shows the relative risk compared with 1 for the low density.

Bold values indicate statistical significance at the P <0.05 level.

* Type 3 Wald test P value for all 3 levels of cell densities for TTR

† Type 3 Wald test P value for all 3 levels of cell densities for DFS

Abbreviations: HR: hazard ratio; CI: confidence interval; TTR: time to recurrence; DFS: disease-free survival; int: intermediate

Table S6. Univariable analysis of clinical and pathology variables associated with time to recurrence (TTR) and disease-free survival (DFS) in 64 most recent patients

	TTR			DFS		
	HR	95% CI	P	HR	95% CI	P
Growth type, n=64 (continuous)	0.89	0.61-1.28	0.521	0.99	0.76-1.28	0.944
Extent of encapsulation, n=64 (continuous)	1.28	0.73-2.24	0.391	0.94	0.63-1.40	0.756
Microvascular invasion, n=64 (yes vs no)	1.95	0.90-4.21	0.089	1.45	0.77-2.72	0.252
Diabetes mellitus, n=64 (yes vs no)	1.98	0.92-4.24	0.080	1.51	0.83-2.76	0.178
Viral hepatitis, n=64 (yes vs no)	0.51	0.14-1.89	0.311	0.39	0.13-1.17	0.092
Gender, n=64 (males vs females)	1.19	0.45-3.14	0.731	0.97	0.46-2.02	0.927
Age, n=64 (continuous)	0.96	0.93-0.99	**0.017**	0.98	0.95-1.01	0.161
Tumor size, n=64 (continuous)	1.00	0.96-1.01	0.349	1.00	1.00-1.01	0.328
Tumor grade (WHO) n=64 (continuous)	0.75	0.36-1.56	0.434	0.69	0.38-1.25	0.219
Tumor grade (Edmondson-Steiner), n=64 (continuous)	0.57	0.24-10.36	0.205	0.67	0.35-1.31	0.242
Architectural grade, n=64 (continuous)	1.01	0.68-1.50	0.969	0.89	0.66-1.22	0.473
Stromal component, n=64 (continuous)	1.01	0.66-1.55	0.970	0.99	0.71-1.39	0.960
Cytological type, n=64 (continuous)	1.25	0.57-2.73	0.580	1.13	0.60-2.11	0.713
Nuclear grade, n=64 (continuous)	0.65	0.39-1.08	0.096	0.72	0.49-1.08	0.110
Nucleolar grade, n=64 (continuous)	0.79	0.49-1.27	0.328	0.90	0.62-1.30	0.567
Necrosis, n=64 (yes vs no)	1.06	0.45-2.46	0.901	1.45	0.77-2.74	0.248
Tumor micronodularity, n=64 (yes vs no)	0.82	0.37-1.79	0.612	1.00	0.54-1.83	0.992
Microsatellites, n=64 (yes vs no)	1.02	0.47-2.21	0.963	1.31	0.72-2.42	0.379
TNM stage, n=64 (continuous)	1.46	0.99-2.15	0.059	1.22	0.86-1.72	0.265
Alpha-fetoprotein, n=46 (continuous)	1.00	1.00-1.00	0.880	1.00	1.00-1.00	0.408
Child-Pugh score, n=63 (A vs B)	1.03	0.04-7.42	0.891	1.18	0.28-4.98	0.827

Table S7. Densities of nucleated profiles of tumor infiltrating lymphocytes (Q_A) in the invasive margin and non-tumor liver associated with time to recurrence and disease-free survival (uni-variable analysis)

	Density	**TTR**			**DFS**		
		HR	95% CI	P	HR	95% CI	P
	**Invasive margin**						
**CD3**, n=65	int vs low	0.98	0.41-2.37	0.963	0.85	0.41-1.77	0.659
0.181*, 0.408#	high vs low	0.39	0.13-1.22	0.105	0.59	0.26-1.32	0.200
**CD8**, n=66	int vs low	0.36	0.15-0.87	**0.023**	0.39	0.19-0.80	**0.010**
**0.019***, **0.008#**	high vs low	0.26	0.09-0.74	**0.012**	0.31	0.14-0.69	**0.004**
**CD20**, n=66	int vs low	0.93	0.40-2.17	0.864	0.96	0.49-1.88	0.894
0.164*, 0.066#	high vs low	0.37	0.12-1.11	0.076	0.39	0.16-0.93	**0.033**
	**Non-tumor liver**						
**CD3**, n=42	int vs low	0.71	0.20-2.56	0.602	0.66	0.26-1.67	0.377
0.630*, 0.687#	high vs low	1.22	0.35-4.26	0.752	0.79	0.31-2.05	0.627
**CD8**, n=43	int vs low	1.74	0.52-5.87	0.369	1.95	0.79-4.84	0.149
0.454*, **0.048#**	high vs low	0.86	0.21-3.48	0.836	0.61	0.20-1.85	0.380
**CD20**, n=42	int vs low	0.48	0.14-1.68	0.249	0.61	0.24-1.56	0.303
0.146*, 0.237#	high vs low	1.51	0.47-4.84	0.485	1.27	0.49-3.29	0.617

For all cells and regions of interest the raw densities of nucleated profiles of CD3+, CD8+ and CD20+ tumor infiltrating lymphocytes per area section (mm^2) were converted into percentiles and then categorized into low (0-25 percentile), intermediate (25-70 percentile), high (70-100 percentile).

Hazard ratios shows the relative risk compared with 1 for the low density.

Bold values indicate statistical significance at the P <0.05 level.

* Type 3 Wald test P value for all 3 levels of cell densities for TTR

Type 3 Wald test P value for all 3 levels of cell densities for DFS

Abbreviations: HR, hazard ratio; CI, confidence interval; TTR, time to recurrence; DFS, disease-free survival

Table S8. Correlation between densities of nucleated profiles of tumor infiltrating lymphocytes (Q_A) in different ROIs (Spearman ρ)

**CD3+ T cells**				
	inn M	out M	PT	NT, *n=42*
TC, *n=67*	ρ=0.79<br>P<0.001	ρ=0.55<br>P<0.001	ρ=0.46<br>P<0.001	ρ=0.34<br>P=0.029
inn M, *n=65*		ρ=0.63<br>P<0.001	ρ=0.46<br>P<0.001	ρ=0.32<br>P=0.041
out M, *n=65*			ρ=0.57<br>P<0.001	ρ=0.31<br>P=0.048
PT, *n=64*				ρ=0.29<br>P=0.067
**CD8+ T cells**				
	inn M	out M	PT	NT, *n=43*
TC, *n=67*	ρ=0.83<br>P<0.001	ρ=0.62<br>P<0.001	ρ=0.41<br>P<0.001	ρ=0.34<br>P=0.025
inn M, *n=66*		ρ=0.62<br>P<0.001	ρ=0.33<br>P=0.008	ρ=0.28<br>P=0.072
out M, *n=66*			ρ=0.60<br>P<0.001	ρ=0.27<br>P=0.085
PT, *n=65*				ρ=0.26<br>P=0.094
**CD20+ B cells**				
	inn M	out M	PT	NT, *n=43*
TC, *n=67*	ρ=0.60<br>P<0.001	ρ=0.22<br>P=0.07	ρ=0.10<br>P=0.432	ρ=0.08<br>P=0.629
inn M, *n=66*		ρ=0.25<br>P<0.041	ρ=0.20<br>P=0.105	ρ=0.14<br>P=0.390
out M, *n=66*			ρ=0.53<br>P<0.001	ρ=0.02<br>P=0.881
PT, *n=65*				ρ=0.09<br>P=0.561

Abbreviations: TC, tumor center; M, tumor invasive margin; PT, peritumor liver; inn M, inner invasive margin; out M, outer invasive margin; NT, non-tumor liver

Table S9. Significant correlations between densities of nucleated profiles of tumor infiltrating lymphocytes (Q_A) in different ROIs (Spearman ρ, P<0.05)

	CD8 TC n=67	CD20 TC n=67	CD8 inn M n=66	CD20 inn M n=65	CD8 out M n=66	CD20 out M n=65	CD8 PT n=65	CD20 PT n=64	CD8 NT n=65	CD20 NT n=64
CD3 TC	0.78	0.68								
CD8 TC		0.67								
CD3 inn M			0.79	0.65						
CD8 inn M				0.62						
CD3 out M					0.61	0.38				
CD8 out M						0.33				
CD3 PT							0.69	0.42		
CD8 PT								0.51		
CD3 NT									0.68	0.48
CD8 NT										0.41

Abbreviations: TC, tumor center; M, tumor invasive margin; PT, peritumor liver; inn M, inner invasive margin; out M, outer invasive margin; NT, non-tumor liver

Table S10. Significant associations between densities of nucleated profiles of tumor infiltrating lymphocytes (Q_A) in different ROIs and clinical and pathology variables

	CD3 TC n=67	CD8 TC n=67	CD20 TC n=67	CD3 inn M n=65	CD8 inn M n=66	CD20 inn M n=65	CD3 out M n=65	CD8 out M n=66	CD20 out M n=65	CD3 PT n=64	CD8 PT n=65	CD20 PT n=64
Age, n=67		0.25*			0.29	0.27		0.28				
Tumor grade WHO, n=67				0.31	0.30			0.33				
Tumor grade, ES n=67	0.35	0.31	0.24	0.25	0.34			0.34				
Nuclear grade, n=67	0.38	0.37	0.32	0.38	0.36			0.34				
Nucleolar grade, n=67	0.28	0.26	0.28									
Tumor size, n=67									-0.33			
TNM stage, n=67									-0.24			
Tumor mi-cronodularity, n=67	P<0.01[#]	P<0.05	P<0.01	P<0.01			P<0.001		P<0.01			
Tumor necrosis, n=67	0.27	0.32			0.29			0.27				
MVI, n=67						P<0.05						
Stromal compo-nent, n=67	0.35	0.39	0.38	0.42	0.33		0.54	0.38	0.25		0.24	
Fibrous encap-sulation, n=67							-0.29		-0.34			

*: Spearman ρ, P<0.05

[#]: P-value of Mann-Whitney U-test

Abbreviations: TC, tumor center; M, tumor invasive margin; PT, peritumor liver; inn M, inner invasive margin; out M, outer invasive margin

Table S11. Significant associations between densities of nucleated profiles of tumor infiltrating lymphocytes (Q_A) in the PT and NT liver and pathology features of non-alcoholic fatty liver disease or chronic hepatitis (Spearman ρ, P<0.05)

	CD3 PT n=64	CD8 PT n=65	CD20 PT n=65	CD3 NT n=43	CD8 NT n=43	CD20 NT n=43
Histopathological features of NAFLD						
Perisinusoidal fibrosis				0.36		0.37
Portal fibrosis				0.35		0.52
Bridging fibrosis				0.33		0.47
Lobular inflammation		0.29		0.40	0.41	0.48
Grade of NASH		0.27				0.30
Stage of NASH				0.35		0.34
Histopathological features of chronic hepatitis						
Grade of chronic hepatitis	0.39	0.37		0.54	0.46	0.73
Stage of chronic hepatitis				0.32		0.46

Abbreviations: NAFLD, non-alcoholic fatty liver disease; NASH, non-alcoholic steatohepatitis; PT. peritumor liver; NT, non-tumor liver
